# CAN CHATGPT IDENTIFY POTENTIALLY INAPPROPRIATE MEDICATIONS (PIM) IN OLDER ADULTS?

**DOI:** 10.1093/geroni/igae098.2988

**Published:** 2024-12-31

**Authors:** Huai Cheng

**Affiliations:** Minneapolis VA Hospital, Playmouth, Minnesota, United States

## Abstract

Reducing prevalent polypharmacy in older adults is critically important in daily practice, but it takes time to do it. Pharmacy care and other practice models can be helpful but costly. ChatGPT passed the USMLE exam and could be used to identify PIM in older adults in daily practice. Based on a case from a published curriculum by the author, ChatGPT was prompted to answer multiple clinical questions with two approaches. First, ChatGPT was prompted to answer four general questions on appropriate use of medications 1) Which of the commonly used 8 drug (s) is (are) PIM and should be avoided in older adults? 2) What is PIM in older adults? 3) How to identify PIM in older adults? 4) What to do with PIM? Second, ChatGPT was prompted to answer additional four specific questions related to the case 1) any drug-drug interactions for this patient? 2) any drug is ineffective for this patient? 3) this patient was taking so many medications. Any way to reduce the number of her medications? 4) Any drug-disease interaction for this patient? The author was the only person to determine the correctness of output from ChatGPT. Overall, ChatGPT can provide the principles of prescribing and de-prescribing for older adults. ChatGPT identified harmful medications and drug-drug interactions, but not drug-disease interactions. This indicated ChatGPT can be helpful in identifying certain PIM, but not drug-disease interactions in older adults. ChatGPT needs to be trained before providers use it to identify all PIM at daily practice.

